# Evaluation of a CdTe semiconductor‐based gamma camera for real‐time dose dosimetry in boron neutron capture therapy

**DOI:** 10.1002/mp.70062

**Published:** 2025-10-08

**Authors:** I‐Huan Chiu, Takahito Osawa, Takehiro Sumita, Kazuhiko Ninomiya, Shin'ichiro Takeda, Tadayuki Takahashi

**Affiliations:** ^1^ Materials Sciences Research Center Japan Atomic Energy Agency Ibaraki Japan; ^2^ Department of Materials Kyushu University Fukuoka Japan; ^3^ Natural Science Center for Basic Research and Development Hiroshima University Higashi‐Hiroshima Hiroshima Japan; ^4^ Fukushima Institute for Research, Education and Innovation (F‐REI) Fukushima Japan; ^5^ Kavli Institute for the Physics and Mathematics of the Universe (WPI) The University of Tokyo Kashiwa Chiba Japan; ^6^ International Center for Quantum‐field Measurement Systems for Studies of the Universe and Particles (QUP, WPI) High Energy Accelerator Research Organization (KEK) Tsukuba Ibaraki Japan

**Keywords:** boron neutron capture therapy, CdTe‐DSD, real‐time dosimetry

## Abstract

**Background:**

Boron neutron capture therapy (BNCT) is a cancer treatment that leverages the nuclear reaction between boron‐10 (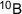) and thermal neutrons to generate high‐energy α particles and 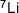 nuclei that selectively destroy cancer cells while sparing healthy tissues. However, BNCT is limited by current dosimetry methods that are incapable of monitoring boron distribution during therapy. An imaging system capable of real‐time dosimetry is essential for optimizing treatment efficacy and minimizing collateral damage.

**Purpose:**

This study aimed to develop a high‐resolution real‐time boron dosimetry system for BNCT by employing a cadmium telluride double‐sided strip detector (CdTe‐DSD). The CdTe‐DSD enables precise mapping of 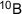 distribution by detecting the 478 keV prompt γ‐rays emitted during the 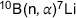 reaction.

**Methods:**

The imaging system was constructed by integrating a CdTe‐DSD with a 2‐mm diameter pinhole collimator. The CdTe‐DSD, comprising a 2‐mm‐thick CdTe semiconductor, has a sensitive area of 32× 32 ${\mathrm{mm}}^{2}$. Neutron irradiation experiments were performed at Japan Research Reactor No. 3 using various boron‐containing samples, including boric acid solutions, powders, and granular boron, with boron masses ranging from 0.02 to 2.00 mg. We implemented the neutron shields using a 5‐mm‐thick 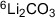 plate and LiF tiles to reduce the background from scattered neutrons during the measurement.

**Results:**

The imaging system successfully detected the 478 keV γ‐ray signal with an energy resolution of 7.3 keV at 511 keV. The reconstructed two‐dimensional 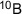 images demonstrate the capability of the CdTe‐DSD to accurately map the 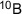 distribution in the samples. Notably, we evaluated the important background contributions from the scattering neutrons in the development of the CdTe‐DSD‐based imaging system. When the scattering neutrons in a neutron experiment hit the CdTe‐DSD, 558 keV γ‐rays of 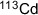 were produced. Under three experimental conditions with different neutron shielding configurations, we found that enhanced neutron shielding considerably reduced the background contribution created by the 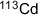 background signal, thereby improving the contrast‐to‐noise ratio (CNR) in the image quality assessment. Upon comparing the condition with minimal neutron shielding to that with maximal shielding, a 14‐fold enhancement in the CNR was observed.

**Conclusions:**

This study demonstrates that a CdTe‐DSD‐based gamma camera is a promising tool for real‐time boron dosimetry in BNCT. The detector's high energy resolution and excellent spatial resolution enable precise detection of 478 keV prompt γ‐rays, facilitating accurate mapping of boron distribution during neutron irradiation. These findings support the developed system's potential to enhance BNCT treatment planning and patient‐specific dose monitoring. Future research will optimize neutron shielding and explore advanced collimator designs to improve sensitivity in clinical settings.

## 1 INTRODUCTION

Boron neutron capture therapy (BNCT) is a cancer treatment that utilizes the nuclear reaction between boron‐10 (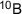) and thermal neutrons to generate high‐energy α particles and 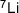 nuclei to selectively destroy cancer cells. It is a tumor cell‐targeted radiotherapy that represents a considerable improvement over conventional radiotherapies with respect to cancer treatment.^1^ 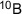 has a large thermal neutron absorption cross‐section  and produces alpha particles (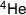) and lithium‐7 (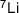) nuclei by capturing a neutron.^2^ This 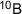 (n, α) 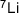 reaction generates charged particles that can be used to destroy cancer cells.^3^ In BNCT, boron‐containing compounds are selectively absorbed by cancer cells and destroyed during neutron irradiation while sparing the surrounding healthy tissue. Despite its potential, BNCT faces substantial challenges, particularly concerning real‐time dosimetry of boron distribution within tumors.^4^ Developing a method for real‐time measurement of boron concentration and spatial distribution is vital for optimizing therapeutic efficacy and minimizing damage to healthy tissues. Currently, boron dosimetry in BNCT typically involves the use of fluoride‐18‐labeled p‐boronophenylalanine (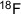
‐BPA) in positron emission tomography (PET) studies to evaluate its accumulation in tumor tissues.^5^ In other words, BNCT practices often rely on pretreatment boron concentration assessments via PET. However, because these assessments do not track the actual boron distribution during neutron irradiation, they may not accurately reflect the true dose distribution at the time of treatment. Implementing a real‐time imaging technique to monitor boron dose distribution throughout the treatment would considerably enhance the accuracy of BNCT.^6^ γ‐rays with an energy of 478 keV are promptly emitted in the 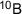 (n, α) 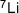 reaction and are known as the prompt γ‐rays of 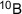. The distribution of the corresponding 478 keV signal directly reveals the actual local position of the reaction; therefore, developing a single‐photon emission computed tomography (SPECT) system^7^, ^8^, ^9^ to measure the 478 keV γ‐ray signal from boron during BNCT treatment offers a practical solution for real‐time BNCT dosimetry. BNCT–SPECT enables the creation of detailed three‐dimensional (3D) spatial images of the dose distribution within the tumor. Several BNCT–SPECT systems using CdTe/CZT semiconductor detectors and scintillators such as ${\mathrm{LaBr}}_{3}$ and GAGG have been introduced.^6^, ^10^, ^11^ However, BNCT–SPECT faces several challenges, such as achieving sufficient energy resolution to separate the 478 keV γ‐rays from the 511 keV positron annihilation background signal and obtaining sufficient spatial resolution (a few millimeters) for BNCT applications. To address these issues associated with BNCT–SPECT applications, we developed a new imaging system using a cadmium telluride double‐sided strip detector (CdTe‐DSD), which offers a high energy resolution of 7.3 keV at 511 keV and an excellent intrinsic positional resolution of 250 μm.^12^

The CdTe‐DSD was developed for hard‐x‐rays and gamma‐ray measurements in astronomy and used in astronomy observations for the ASTRO‐H mission owing to its high performance.^13^, ^14^, ^15^, ^16^, ^17^ The CdTe‐DSD was constructed with a 0.75‐mm‐thick CdTe crystal,^18^ offering approximately 40% detection efficiency for 100 keV γ‐rays,^13^ with an energy resolution of less than 2 keV full width at half maximum (FWHM) at 122 keV. In recent years, CdTe‐DSDs have been used for 3D elemental analysis in quantum beam science,^19^ verifying their suitability for application in SPECT systems. Additionally, CdTe‐DSDs combined with multi‐pinhole and parallel‐hole collimators have been employed for SPECT in medical analysis,^20^, ^21^ and have achieved a spatial resolution of less than 500 μm. CdTe‐DSD‐based SPECT systems have shown the ability to monitor real‐time activity changes in tumors through time‐activity curves. The recent development of CdTe‐DSDs with a 2‐mm‐thick CdTe crystal has led to improved accuracy and detection efficiency for high‐energy (>100 keV) signals. These CdTe‐DSDs maintain a positional resolution of a few hundred micrometers, thereby offering high detection efficiency in the high‐energy region with an energy resolution of 7.3 keV at 511 keV.^12^ High positional resolution enables a more precise assessment of the boron dose distribution in the tumor, while high energy resolution helps distinguish the 478 keV γ‐rays from the 511 keV positron annihilation background signal. Given the success of CdTe‐DSD‐based SPECT systems in various fields and the improved detection sensitivity for 478 keV γ‐rays with the 2‐mm‐thick CdTe crystal, CdTe‐DSDs are ideal for the development of BNCT–SPECT.

This study investigated the feasibility of using a 2‐mm‐thick CdTe‐DSD for applying BNCT–SPECT in measuring boron distribution during neutron irradiation. The imaging system for BNCT–SPECT was designed by integrating a 2‐mm‐diameter pinhole collimator with the CdTe‐DSD. Neutron experiments were conducted at the Japan Research Reactor No. 3 (JRR‐3) facility. The imaging capabilities of the CdTe‐DSD were evaluated via a neutron experiment considering factors potentially affecting image quality. Various boron‐containing compounds, including boric acid solutions of different concentrations, boric acid powders, and pure boron, were used in this study. The performance of the imaging system was quantitatively analyzed to assess its accuracy and image quality.

## 2 METHODS

### 2.1 Design of the imaging system

**FIGURE 1:**
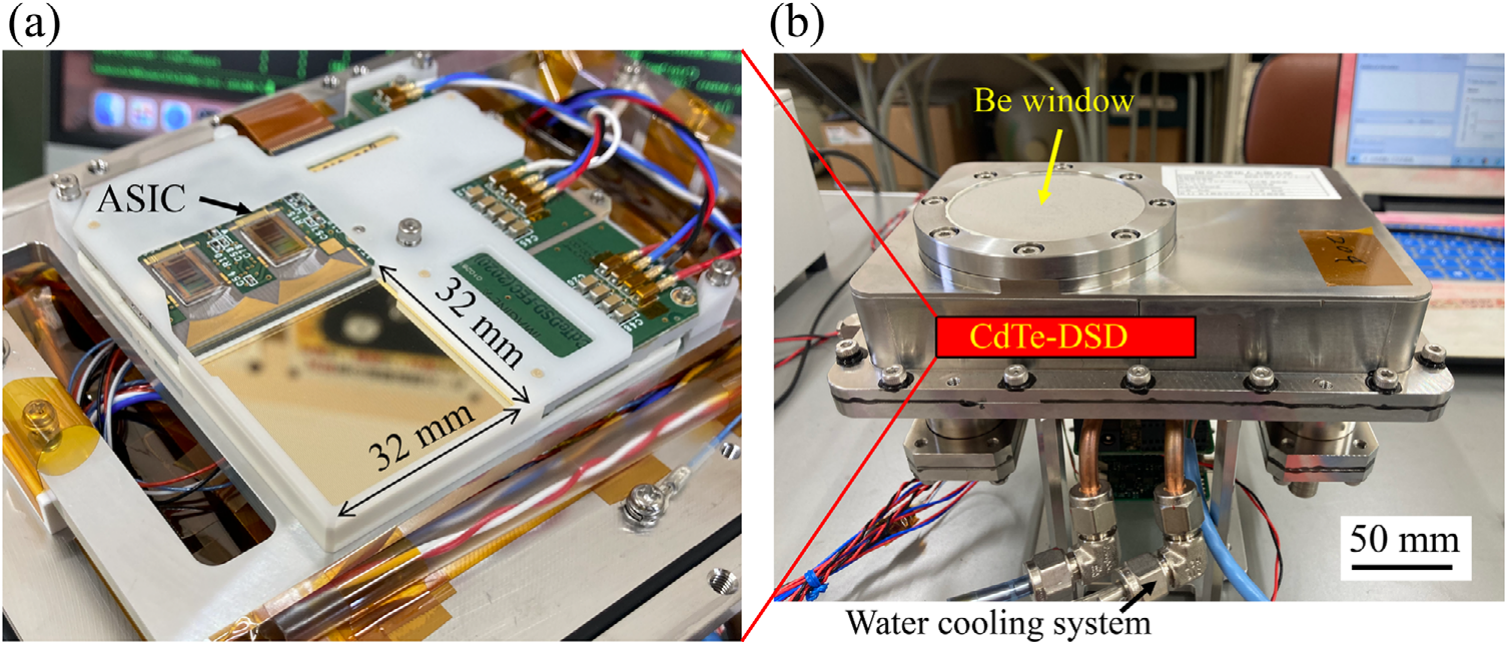
(a) Photograph of the CdTe‐DSD with the designed ASIC. (b) Photograph of the integrated Al housing of the water‐cooling system, with the CdTe‐DSD installed inside the housing. ASIC, application‐specific integrated circuit; CdTe‐DSD, cadmium telluride double‐sided strip detector.

Figure 1 shows photographs of the CdTe‐DSD (left) and an Al housing (right) used in this study. The sensitive area of the CdTe‐DSD measures 32 mm × 32 mm. The detector features a sensitive area with 128 Al and 128 Pt strip electrodes, arranged with a 250‐μm strip pitch and a 50‐μm gap on the anode and cathode sides, forming a double‐sided strip structure. When an x‐ray or γ‐ray photon is absorbed by the CdTe crystal, electron–hole pairs are generated and move towards the anode and cathode under the applied high bias voltage. This bias voltage is essential for creating an electric field that efficiently collects the charges.^17^ In this study, the Al side, corresponding to the anode, was maintained at 0 V, while the Pt side, corresponding to the cathode, was maintained at 500 V. The orthogonal electrodes on either side provided simultaneous two‐dimensional positional information regarding the incident location within the CdTe‐DSD and the energy of the detected signal. The double‐sided strip structure of the CdTe‐DSD enabled imaging with 16,384 pixels while utilizing only 256 readout channels (2 × 128 channels). A compact readout system was realized using specially designed ASICs with 64 readout channels.^22^ Each side's 128 channels were connected to two ASICs, forming the readout system. The detected signal underwent integration and shaping, followed by analog‐to‐digital conversion, and was output as digital complementary metal‐oxide‐semiconductor (CMOS) data.^23^ The CdTe‐DSD functioned as a Schottky‐diode detector. The Schottky CdTe diode faces polarization issues when operating under high bias voltage,^24^ which can degrade its performance. To address this problem, the operational temperature of the CdTe‐DSD was maintained below $-20^{\circ }C$, thereby ensuring high performance and stability. Maintaining the temperature in this range reduced polarization effects and improved the overall reliability of the CdTe‐DSD.^25^ A cooling setup, comprising Peltier elements and a water‐cooling system, was implemented to maintain the CdTe‐DSD detector below $-20^{\circ }C$. Consequently, the detector achieved an energy resolution of > 2% (FWHM/energy) over an energy range of tens to hundreds of keV. The detector was housed in a custom‐designed Al housing (Figure 1, right) to achieve temperature stability. To prevent water vapor condensation on the CdTe‐DSD at the low temperature of $-20^{\circ }C$, which could damage the detector, the housing was filled with dry nitrogen ($N_{2}$). A Be window was installed in front of the Al housing to minimize attenuation of the incoming radiation, thereby ensuring accurate and reliable detection results from the CdTe‐DSD. A pinhole collimator with a 2‐mm‐diameter hole was integrated in front of the Be window to measure the projection images of boron samples. The tungsten‐made collimator with a diameter and thickness of 63 and 8 mm, respectively, was installed at a distance of 48 mm from the CdTe‐DSD. The transmission of 500 keV γ‐rays outside the collimator was as low as 10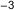 and the field of view (FOV) was limited to $67.5^{\circ }$ from the “knife‐edge” geometry of the pinhole configuration.

### 2.2 Neutron source at japan research reactor no. 3 (JRR‐3)

The JRR‐3 facility at the Japan Atomic Energy Agency in Tokai‐mura, Japan, supports scientific and industrial applications. This research reactor is renowned for its high neutron flux, which is essential for advanced studies in materials science, biology, and nuclear physics. The prompt γ‐ray analysis (PGA) apparatus at beam port T1‐4‐1 in the JRR‐3 is used for nondestructive elemental analysis owing to its exceptionally low background environment that produces high‐quality analytical data, particularly during boron analysis. High‐purity germanium (HPGe) detectors are used to detect and measure the energy spectra of the emitted prompt γ‐rays. The JRR‐3 offers a stable and high‐flux thermal neutron source with an energy of 0.025 eV for PGA, delivering 1.8 × 10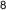 neutrons/${\mathrm{cm}}^{2}\cdot s$ with a beam size of 2 × 2 ${\mathrm{cm}}^{2}$.^26^ The internal structural materials of the PGA apparatus comprise polytetrafluoroethylene (PTFE) and LiF tiles, considerably reducing the generation of prompt γ‐rays from the apparatus itself. Samples are placed within the neutron beam path inside a PTFE sample chamber, allowing interactions between the neutrons and sample elements. The PGA apparatus considerably reduces the background from the scattering neutrons by filling the sample chamber with ${\mathrm{CO}}_{2}$. These measures help maintain a low‐background environment in the PGA system, enabling accurate quantification of elements in the samples. Given the high accuracy of PGA quantification using the HPGe detector, this experiment utilized its measurement data to estimate the prompt γ‐ray activity from 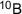 in the samples. Additionally, a CdTe‐DSD was installed above the PGA apparatus to capture the boron distribution in the sample.

### 2.3 Experimental setup

**FIGURE 2:**
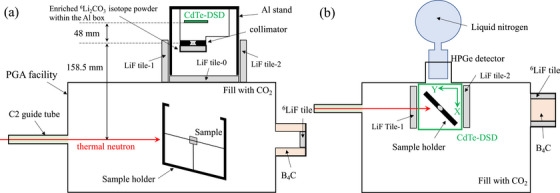
(a) Schematic side view of the experimental setup, featuring the CdTe‐DSD installed above the PGA apparatus. (b) Top view of the experimental setup. CdTe‐DSD, cadmium telluride double‐sided strip detector; PGA, prompt gamma‐ray analysis.

Figure 2a shows the schematic side view of the experimental setup with the CdTe‐DSD and the samples in the PGA apparatus; photographs of the setup are presented in Figure S1. The CdTe‐DSD was installed above the PGA apparatus to capture signals from the boron sample during neutron irradiation. The distances from the pinhole collimator to the CdTe‐DSD and the sample were 48 and 158.5 mm, respectively. Panel b presents the schematic top view of the experimental setup with the HPGe detector. The X and Y axes for the reconstructed image of the CdTe‐DSD are indicated by green arrows.

**FIGURE 3:**
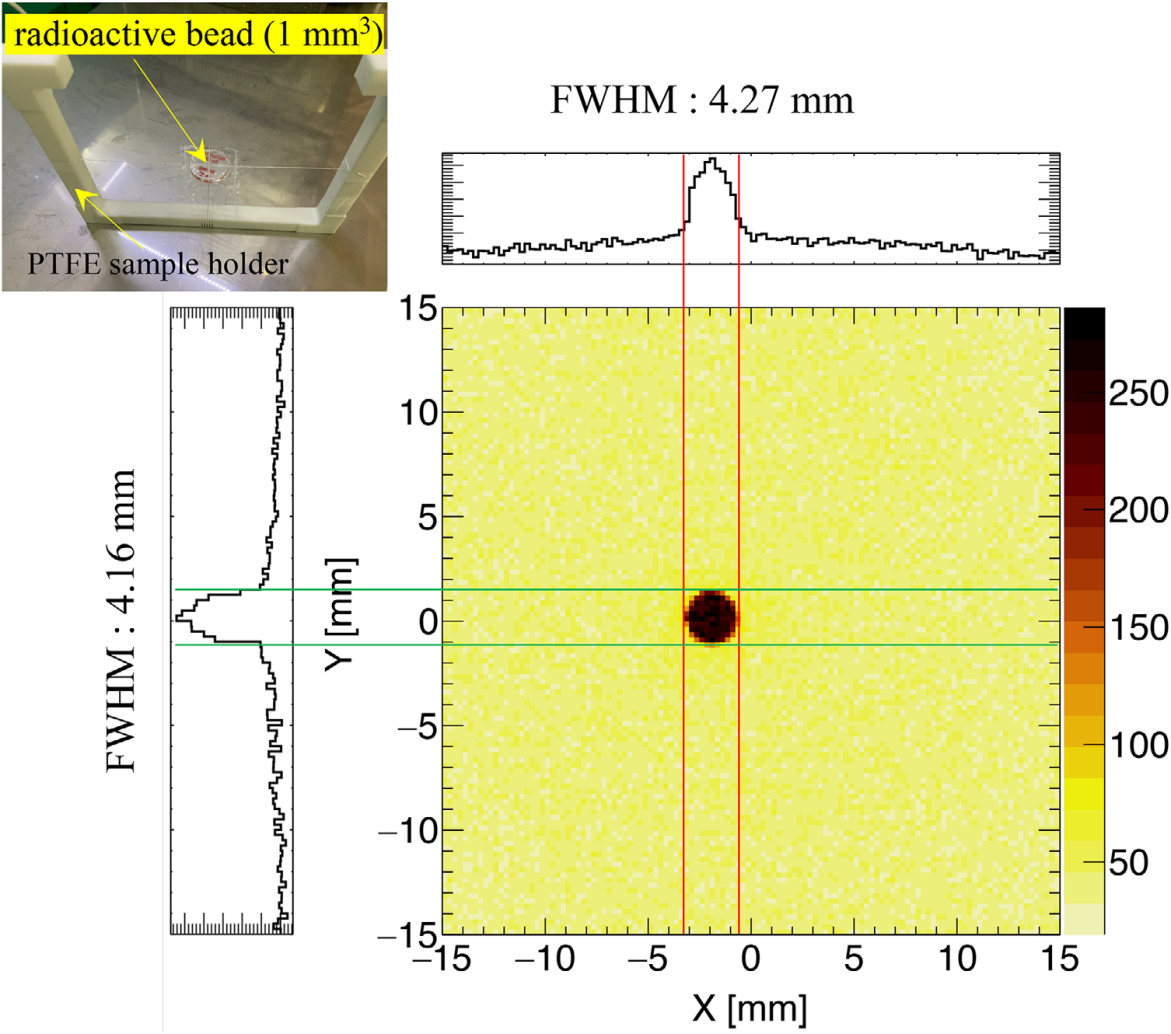
(Upper left) Photograph of the 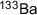 radioactive isotope (RI) source featuring a radioactive bead of 1 ${\mathrm{mm}}^{3}$, mounted on a PTFE sample holder. The reconstructed CdTe‐DSD image with the projection distributions along the *X* and *Y* axes indicates the location of the signal from the 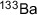 RI source. The 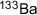 images show a FWHM of approximately 4 mm in both directions. CdTe‐DSD, cadmium telluride double‐sided strip detector; FWHM, full width at half maximum; PTFE, polytetrafluoroethylene; RI, radioactive isotope.

First, we used a 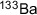 radioactive isotope (RI) source with an intensity of 3.85±0.193 MBq to quantitatively assess the spatial resolution of the imaging system. The 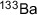 RI source is encapsulated in a bead approximately 1 mm in diameter, which is then placed inside a solid plastic capsule with a diameter and thickness of 25 and 3 mm, respectively. The upper left corner of Figure 3 presents a photograph of the 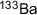 RI source, which is fixed to the PTFE sample holder using PTFE thread. Radioactive source measurement was performed over a period of 3699 s. We reconstructed the image of the 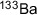 RI source, with projection distributions along both the *X* and *Y* axes (Figure 3). Gaussian fitting was applied to the projection distributions to determine the corresponding mean and FWHM. The means were −1.89 mm on the *X*‐axis and 1.97 mm on the *Y*‐axis. The corresponding FWHMs were 4.27 ± 0.12 mm and 4.16 ± 0.09 mm, respectively. This result shows that the position resolution of the imaging system is approximately 4 mm.

Next, the scattered neutron background considerably increases when neutrons interact with the sample or the experimental setup in a neutron exposure experiment. When the scattered neutrons directly hit the CdTe‐DSD, they interact with the 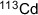 within the CdTe crystal, producing prompt γ‐rays at energies of 558 keV. Given that 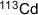 has a large interaction cross‐section with neutrons, the intense 558 keV γ‐rays produced by 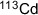 are a dominant background in the energy spectrum. Although the CdTe‐DSD has high enough energy resolution to distinguish the 478 keV signal from the 558 keV peak, the 558 keV photons still produce continuous background near 478 keV due to Compton scattering. This Compton continuum overlaps with the 478 keV signal and affects the quantitative analysis of the boron sample. Therefore, investigating the impact of scattered neutrons on boron measurements using the CdTe‐DSD is crucial. We placed a 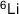 shadow plate in front of the pinhole collimator to prevent neutrons from hitting the detector. The 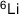 shadow plate consists of an Al container (140 mm ×110 mm, 5‐mm thick) with a 0.5‐mm‐thick lid. The Al container was filled with 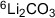 powder. Because 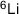 has a considerably high neutron absorption efficiency,^27^ the 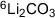 shield greatly reduces (by more than 99%) the background generated by the neutrons scattered from the sample. Additionally, the 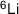 shadow plate negligibly affects the 478 keV γ‐ray signal from the sample. The neutrons scattered by the PGA apparatus and beam stopper from other directions still contribute to the background. To assess the effect of the scattered neutrons on the CdTe‐DSD image, we employed three experimental conditions (Experiments 1, 2, and 3), gradually increasing neutron shielding in each condition (Figure 2).

1. Experiment 1: The experiment was conducted using only the 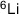 shadow plate placed in front of the pinhole collimator.
2. Experiment 2: A 10 mm‐thick LiF plate (LiF Tile‐0 and LiF Tile‐1 in Figure 2a) was added around the imaging system to reduce the background generated by the neutrons scattered by the PGA apparatus.
3. Experiment 3: To address the neutrons that pass through the sample and strike the neutron beam stopper downstream, causing scattered neutrons to hit the CdTe‐DSD, we added another 10‐mm LiF plate (LiF Tile‐2 in Figure 2a) between the CdTe‐DSD and the beam stopper.

### 2.4 Boron samples

In the sample selection for BNCT‐SPECT studies, the Jaszczak phantom—which contains substantial amounts of water—is often used to evaluate imaging performance. This is because using such a sample allows for the assessment of intense background signals that originate from water‐rich tissues in the human body, where hydrogen has a high neutron absorption cross‐section and emits prompt γ‐rays at 2223 keV upon neutron capture. In this study, we considered the effects of neutron energy in the PGA. The neutron energy in the PGA is 0.025 eV, which is significantly lower than the 0.5 eV < E < 10 keV neutron beams typically used in conventional BNCT.^28^ Consequently, for a water‐rich sample, the low‐energy neutrons in the PGA are primarily absorbed by the hydrogen in the water and cannot reach the 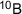 in the deeper regions of the sample. This limitation affects our assessment of the imaging capability of the CdTe‐DSD for boron. Therefore, we disregarded the effect of the hydrogen background and focused on evaluating the imaging capability of the CdTe‐DSD for detecting boron in a low‐energy neutron experiment. We selected samples with negligible hydrogen concentration to minimize the impact of hydrogen on boron measurements using the CdTe‐DSD. Thus, the intensity of the boron signal was directly correlated with the 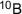 concentration in the sample. Granular solid boron (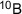), boric acid powders ($H_{3}{\mathrm{BO}}_{3}$), and boric acid solutions ($H_{3}{\mathrm{BO}}_{3}$ + $H_{2}O$) were used to evaluate the performance of the imaging system under three experimental conditions. The boron solutions with different concentrations were prepared by diluting Merck TraceCERT 01932 with ultrapure water (18.2 MΩ·cm, Millipore Direct‐Q UV 3). The boric acid powders were obtained by drying Merck TraceCERT 01932, while the granular solid 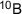 was in its pure elemental form. For boron masses ranging from 0.02 to 2.00 mg, the samples and their corresponding irradiation times under the different experimental conditions are listed in Table 1. Because sample No. 4 had the highest boron mass, an irradiation time of 2 hours was sufficient to obtain the necessary statistics for image quality evaluation. Photographs of samples Nos. 1–7 and the corresponding neutron beam profile at the PGA are provided in Figure S2.

**TABLE 1:** Samples under each experimental condition with their mass and irradiation times.

Experiment	Sample	Form	Total mass (mg)	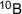 concentration (mg)	Irradiation time (s)
1	No.1	Solution	200	0.02	72000
	No.2	Solution	200	0.20	64800
	No.3	Solution	200	1.00	52041
	No.4	Solution	200	2.00	7199
2	No.5	Powder	1.15	0.20	61199
	No.6	Powder	2.29	0.40	17999
3	No.7	Granular	0.66	0.66	68818

**FIGURE 4:**
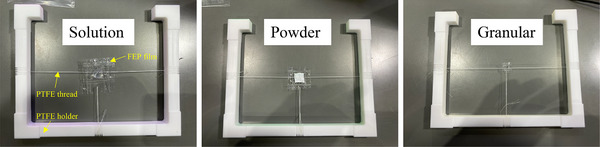
Photographs of sample No. 4 (left), No. 6 (center), and No. 7 (right) encapsulated in FEP film and mounted on a PTFE sample holder using PTFE threads. FEP, fluorinated ethylene propylene; PTFE, polytetrafluoroethylene.

Figure 4 shows sample Nos. 4, 6, and 7 from left to right. The sample encapsulated in fluorinated ethylene propylene (FEP) film was mounted at the center of the PTFE sample holder using PTFE threads. The γ‐rays emitted from PTFE and FEP are typically negligible, as both C and F have low neutron cross‐sections. Furthermore, the sample size was controlled to fit within the neutron beam size of 20×20 ${\mathrm{mm}}^{2}$ because samples larger than this size would be partially non‐irradiated, affecting the accuracy of elemental quantification.

## 3 RESULTS

### 3.1 Calibration curve of boron activity

**FIGURE 5:**
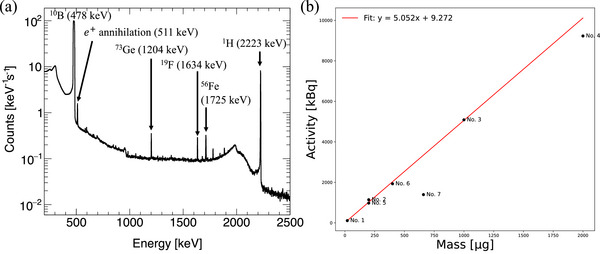
(a) Energy spectrum recorded by the HPGe detector at the PGA during the measurement of sample No. 4. (b) Calibration curve illustrating the relationship between boron mass and the intensity of boron prompt γ‐rays. HPGe, High‐purity germanium; PGA, prompt gamma‐ray analysis.

Figure 5a shows the energy spectrum of sample No. 4 from the HPGe detector measurement. The 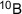 signal at 478 keV is clearly observed, accompanied by several weaker backgrounds from $e^{+}$ annihilation, 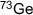, 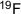, 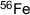, and 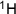. The 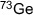 and 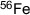 signals originate from scattered neutrons hitting the Ge crystal of the HPGe detector and PGA apparatus. The 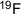 signal originates from the FEP film, and the 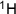 stems from trace water present in the sample. The activities of the 478 keV 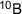 γ‐ray signal from samples Nos. 1–7 were estimated using HPGe detector measurements. The HPGe detector's efficiency for detecting 478 keV γ‐rays is 0.0143%, as determined via a 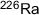 RI source experiment. The intensity of the 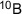 γ‐ray signal from each sample was then calculated as $\frac{N_{B}/t}{0.0143\%}$, where $N_{B}$ represents the counts recorded by the HPGe detector and t represents the measurement time. The calculated intensities of the 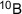 γ‐ray signal are summarized in Table 2.

**TABLE 2:** Summary of activities, ROI intensities, background region (bkg) intensities, and corresponding CNR values.

Experiment	Sample	Activity (kBq)	ROI (cnt./hour)	bkg (cnt./hour)	CNR
1	No.1	110 ± 0.1	2700 ± 52.0	2640 ± 51.4	0.0020 ± 0.053
	No.2	1140 ± 0.3	2950 ± 54.3	2920 ± 54.0	0.039 ± 0.059
	No.3	5090 ± 0.9	3530 ± 59.4	3390 ± 58.3	0.20 ± 0.059
	No.4	9230 ± 2.9	3650 ± 60.4	2820 ± 53.1	0.53 ± 0.054
2	No.5	976 ± 0.3	2000 ± 44.7	1820 ± 42.6	0.14 ± 0.058
	No.6	1930 ± 0.9	2750 ± 52.5	2330 ± 48.3	0.40 ± 0.056
3	No.7	1390 ± 0.4	1580 ± 39.8	1130 ± 33.6	0.56 ± 0.064

Abbreviations: CNR, contrast‐to‐noise ratio; ROI, region of interest.

Figure 5b displays the calibration curve for the 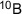 γ‐ray signal activity and the 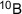 concentration (μg) of the sample. The calibration curve is established using a simple linear fit (red line). It excludes the results for sample Nos. 4 and 7 owing to the following considerations. First, the result for sample No. 4, which contained a 2000 μg 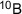 concentration, did not follow the expected linear trend. This deviation is attributed to the high dead time of the HPGe detector which affects the quantitative evaluation of boron activity. Analysis of the fitting results shows that part of the observed deviation from the expected linear relationship is also influenced by the overall system dead time, affecting the measured counts. Thus, factors related to the detector system must be considered when interpreting the relationship between boron content and γ‐ray intensity. The result for sample No. 7 also did not follow a linear trend. Studies have indicated that under extremely high boron concentrations (e.g., in granular form), the neutron shielding effect causes these samples to deviate from the calibration curve established with liquid samples. This neutron shielding phenomenon depends not only on neutron energy but also on sample size and material density.^29^, ^30^ Consequently, sample No. 7 also shows deviation from a linear relationship, with γ‐ray self‐absorption further contributing to this deviation. Considering the aforementioned factors, sample Nos. 4 and 7 were not used in establishing the calibration curve. These complexities necessitate the use of simulations in BNCT to correct the measured data and ensure accurate results.

### 3.2 Spectral analysis and imaging

**FIGURE 6:**
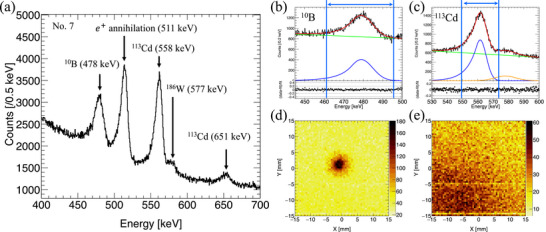
(a) Energy spectra for sample No. 7 measured under Experiment 3. (b) Fitting result for the energy region of 445–500 keV, corresponding to the 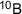 signal. (c) Fitting result for the energy region of 530–600 keV, corresponding to the 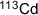 signal. (d) Reconstructed 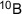 image. (e) Reconstructed 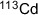 image.

Figure 6a shows the energy spectrum from 400 to 700 keV observed by the CdTe‐DSD for sample No. 7. The 478 keV prompt γ‐ray from 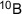 in the sample is observed. Additionally, the 511 keV annihilation γ‐ray from positron–electron interactions is present. Several distinct peaks are observed in the background, corresponding to 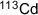 (558 and 651 keV), and 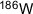 (577 keV). The background signals from 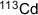 and 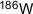 originate from scattered neutrons that directly hit the CdTe crystal and collimator. Panel b presents the fitting result for the 478 keV 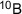 signal. The peak of the 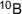 prompt γ‐ray was modeled using a Crystal Ball function,^31^ which comprises a Gaussian core and an asymmetric power‐law tail (depicted by the blue line). A first‐order polynomial function (pol1) was used to model the continuous background (green). Therefore, the 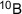 energy spectrum was fitted with the function $f_{\mathrm{crystalball}}\times I_{B}\;+\;f_{\mathrm{pol}}1\times I_{\mathrm{BGD}}$, where $I_{B}$ and $I_{\mathrm{BGD}}$ represent the intensities of the 478 keV 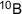 peak and the continuous background ($I_{\mathrm{BGD}}$). Panel c shows the fitting for the 558 keV 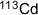 signal, with a minor contribution from the 577 keV 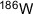 signal. The fitting function (red) includes the 558 keV 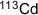 signal (blue), 577 keV 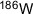 signal (orange), and the continuous background (green). We utilized signals from a specific energy range to reconstruct the images corresponding to the 478 keV 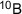 and 558 keV 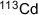 signals. The signals were selected using the energy range $\mu_{B(\mathrm{Cd})}\pm 3\times \sigma_{B(\mathrm{Cd})}$ (blue double arrow line), where $\mu_{B(\mathrm{Cd})}$ and $\sigma_{B(\mathrm{Cd})}$ represent the mean and standard deviation of the fitting result for 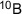 (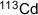), respectively. The reconstructed images for the 478 keV 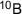 and 558 keV 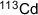 signals are displayed in Panels d and e, respectively. Panel d confirms successful identification of the 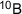 distribution in the sample. Panel e shows a strong 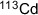 γ‐ray background signal from the (−*X*, −*Y*) direction, which corresponds to the area of the imaging system that is not covered by the LiF tile. Scattered neutrons from this direction interact with the CdTe crystal, resulting in an intense 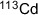 signal in the lower‐left region of the image. Additionally, two horizontal lines around *Y* = −5 mm and *Y* = −15 mm can be observed. These are due to signal loss caused by dead channels in the CdTe‐DSD and consistently appear in all images obtained with the CdTe‐DSD.

### 3.3 Image comparison

**FIGURE 7:**
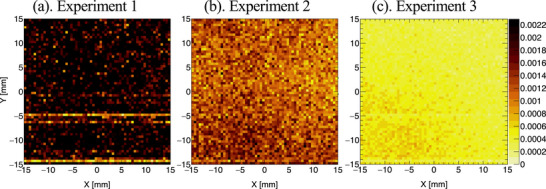
Images of the 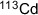 background signal acquired in Experiments 1–3.

Figure 7 shows a comparison of the 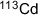 distributions in Experiments 1–3, each conducted under different LiF shielding conditions. The intensity is normalized to counts per second (cps). We found that the intensity of the 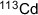 background signal is strongly related to neutron shielding. The 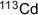 distributions correspond to the areas of the CdTe‐DSD that are hit by scattered neutrons. Panel c shows that the 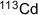 background is considerably suppressed by increasing the LiF shield around the detector.

**FIGURE 8:**
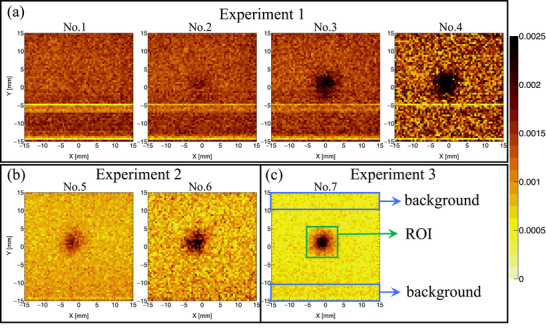
Reconstructed images using the 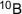 signal from samples Nos. 1–7 in Experiments 1, 2, and 3. The region of interest (ROI) and the background region are defined for the subsequent discussion of image quality.

Figure 8 shows the 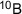 images with the intensity normalized to cps. Although the signals from 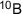 and 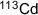 are clearly separated in the energy spectrum, the Compton scatter background from the 558 keV signal still substantially affects the resolution of the 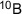 images. The 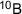 images are notably impacted by the 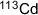 signal. By comparing the measured 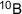 images from Experiment 1 (No. 1–4), Experiment 2 (No. 5–6), and Experiment 3 (No. 7), we clearly observe an increase in the signal intensity at the center, which correlates with the increase in 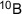 concentration. Additionally, the contrast between the central signal area and surrounding background is strongly influenced by the intensity of the 558 keV 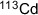 signal. For instance, samples Nos. 2 and 5 exhibit the same 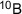 concentration of 0.2 mg, but sample No. 5 shows better contrast due to the experiment being conducted in a lower 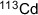 background environment. Therefore, the LiF shielding for the scattered neutrons is crucial when using the CdTe‐DSD for 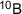 imaging in BNCT. To quantitatively assess the impact of LiF shielding on 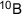 imaging quality, we defined a region of interest (ROI) and a background region, as illustrated in Figure 8c and detailed in the discussion section.

## 4 DISCUSSION

We employed the HPGe detector and CdTe‐DSD based imaging system to measure the 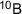 signal from the samples at the JRR‐3. For typical spherical tumors with diameters ranging from 1 to 3 cm and a mean 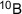 concentration of 50 ppm employed in BNCT clinical practice,^1^ the corresponding 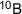 mass is 0.03–0.7 mg. The sample masses used in this study, ranging from 0.02 to 2.00 mg, are consistent with this typical 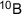 mass range for BNCT clinical practice. The activity of the 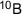 signal, along with the corresponding standard errors for each sample, is shown in Table 2 and was evaluated based on the HPGe detector measurement data. The feasibility of obtaining a 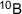 distribution was assessed using CdTe‐DSD data. The CdTe‐DSD demonstrated a high energy resolution of 7.3 keV at 511 keV, enabling it to separate the 478 keV 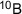 signal from the backgrounds of 511 keV annihilation γ‐ray and 558 keV 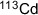 signal. Moreover, the charge collection efficiency for a CdTe semiconductor detector depends on the depth of the γ‐ray interaction. High‐energy (>100 keV) γ‐rays tend to interact deeper within the detector, producing more pronounced low‐energy tails.^12^ We used a Crystal Ball function to accurately quantify the 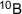 prompt γ‐ray with energy of 478 keV. The continuous background components from the other Compton effect background near the 478 keV region was modeled using a pol1 function. The CdTe‐DSD combined with a 2‐mm‐diameter pinhole collimator was used to obtain the projection image of the 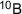 signal. The spatial resolution of the imaging system is relative to the intrinsic positional resolution (250 μm) of the CdTe‐DSD and the collimator parameters, including the length of the hole and the size of the diameter. The spatial resolution was experimentally confirmed to be approximately 4 mm using a 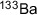 RI source with a volume of 1 ${\mathrm{mm}}^{3}$.

The quality of the 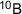 image is considerably impacted by the background contribution. First, this experiment explored the effect of background noise from the 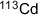 gamma ray with an energy of 558 keV, caused by scattered neutrons hitting the CdTe‐DSD. Although the CdTe‐DSD has high energy resolution to distinguish the 478 keV 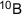 signal from the 558 keV 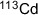 background signal, the Compton scatter background generated by the 558 keV 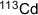 signal considerably increases the continuous background component in the 478 keV energy region. By adding the LiF shields to block the scattered neutrons from the CdTe‐DSD, we effectively reduced the 558 keV background signal. Figure 7 shows a comparison of the 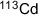 distributions under different experimental conditions. We effectively reduced the 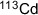 background, which improved the contrast between the 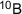 signal and surrounding background.

**FIGURE 9:**
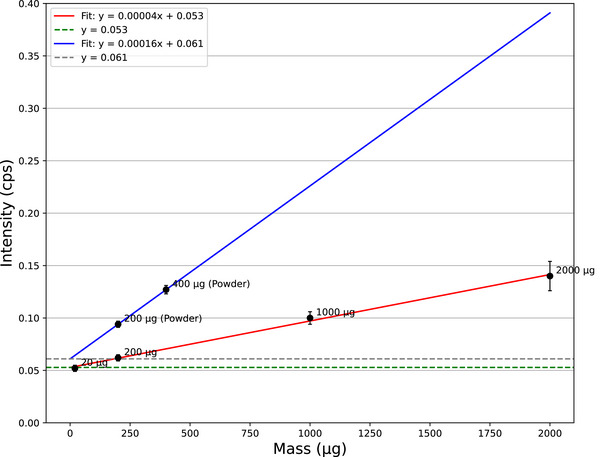
Calibration curve of 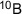 mass and prompt γ‐ray intensity of the CdTe‐DSD. CdTe‐DSD, cadmium telluride double‐sided strip detector.

Next, we investigated the background created by the beam stopper in the PGA apparatus (Figure 2). The beam stopper in the PGA apparatus is made of $B_{4}C$ with the LiF tiles, as shown in Figure S1. When neutrons pass through the sample and are stopped by the beam stopper, $B_{4}C$ produces 478 keV 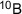 signals from the downstream side of the beam. Because the HPGe detector in the PGA facility is equipped with lead shielding to block these signals from the beam stopper, no 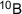 background signals are detected by the HPGe detector. However, the CdTe‐DSD in this experiment lacks such shielding, resulting in the beam stopper contributing to the 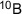 background signals detected by the CdTe‐DSD. We observed 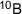 background signals in the calibration curves relating mass to intensity for Experiments 1 and 2 (Figure 9). In these experiments, the strong 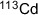 background originating from scattered neutrons complicated the assessment of the spatial distribution of the 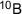 background signals in the images. As the 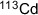 signal decreased in Experiment 3 (Figure 8c), the 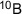 background signals from the downstream side of the neutron beam become more apparent. We found that the intensity in the background region on the −*Y* side of the image (downstream) is stronger than on the +*Y* side (upstream). We calculated the intensities in the −*Y* direction (−15 < *y* < −7, $I_{\mathrm{downstream}}$) and the +*Y* direction (7 < *y* < 15, $I_{\mathrm{upstream}}$). The $\frac{I_{\mathrm{downstream}}}{I_{\mathrm{upstream}}}$ ratios were found to be 1.14 in Experiment 3. This intensity difference confirms that the 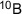 background signal from the downstream side is stronger. In addition, this phenomenon was further verified via a blank measurement using only the sample holder in Experiment 3, as shown in Figure S3. We observed 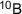 background signals from the downstream side in both the energy spectrum and the image. In this study, 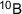 background signals from the beam stopper cannot be completely eliminated. Therefore, when quantitatively assessing the imaging quality in this experiment, accounting for this additional background contribution is important.

We evaluated the image quality based on the data presented in Figure 8 by considering the 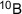 intensity from the sample alongside the 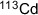 background signal and 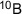 background signal from the beam stopper. The 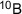 signal from the sample, passing through the pinhole collimator, generates a strong circular signal at the center of the image. The 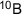 background signal from the neutron stopper and 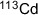, after undergoing considerable scattering, is distributed across the entire image. Therefore, we can quantitatively assess quality by evaluating an image with respect to the relative positions of the sample and detector. Signal‐to‐noise ratio (SNR) is widely used in evaluating medical images such as MRI and CT.^32^ However, in this study, the contrast between the 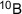 signal and background is an important factor affecting image quality. We used the contrast‐to‐noise ratio (CNR) instead of SNR as CNR more accurately reflects the impact of noise and serves as a meaningful indicator of image quality under different experimental conditions.^33^, ^34^ The ROI is defined based on the spatial location of the boron signal within the image. This spatial area of ROI along the *X*‐ and *Y*‐axes was determined by applying Gaussian fitting to the circular boron signal, with the background region defined as an area of equal size surrounding the ROI, as illustrated in Figure 8c. Table 2 presents the intensities (in counts per hour) for both the ROI and the background region. The detected intensities corresponds to a one‐hour neutron irradiation at the PGA, during which $∼10^{11}$ neutrons with the energy of 0.025 eV are delivered. The average intensities of pixel values within the ROI and the background region are represented by $I_{\mathrm{ROI}}$ and $I_{\mathrm{bkg}}$, respectively. Thus, the CNR is calculated as ($I_{\mathrm{ROI}}$‐$I_{\mathrm{bkg}}$)/$\sigma_{\mathrm{bkg}}$,^35^, ^36^ where $\sigma_{\mathrm{bkg}}$ is the standard deviation of the intensity for the background region. This approach allows for a comparison of the central signal intensity with the surrounding background contrast, taking into account the influence of the background signal on the images. The rightmost column in Table 2 lists the calculated CNR values. The CNR increases with the amount of boron in the sample. Moreover, we can clearly observe considerable improvements in CNR as the 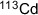 background decreases. As LiF shielding increased in Experiments 1–3, the background intensity considerably decreased and higher CNR values were observed. Consequently, the presence of the LiF shield is crucial for obtaining clearer boron images by reducing the Compton scatter 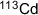 background when using the CdTe‐DSD in BNCT. This study presents experimental validation conducted at the JRR‐3, confirming the feasibility and accuracy of the CdTe‐DSD in detecting prompt γ‐rays. The system's high sensitivity and spatial resolution enabled accurate elemental analysis, making it suitable for BNCT applications. However, the current CdTe‐DSD system still has several challenges for clinical application. First, the samples used in this experiment did not account for background contributions from water content, which generates prompt γ‐ray background in actual BNCT patients. Moreover, the neutron beam at the JRR‐3, with an energy of 0.025 eV, differs significantly from that of accelerator‐based BNCT (AB‐BNCT),^37^ where neutrons have energies of a few keV. Although AB‐BNCT systems provide higher neutron fluxes, the cross‐section of the 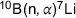 reaction at 1 keV is approximately 0.5% of that at thermal neutron energy (0.025 eV), as used in JRR‐3.^38^ As a result, we expect a much lower boron signal intensity under AB‐BNCT conditions. The background environment in clinical AB‐BNCT is also expected to differ from that of JRR‐3, and its impact on CNR performance needs to be evaluated. Furthermore, additional clinical factors such as patient motion and heterogeneous boron uptake in vivo were not considered in this study. Therefore, we need to assess the performance of the CdTe‐DSD system under realistic AB‐BNCT conditions, along with further system optimization, such as increasing the CdTe crystal thickness and improving the data acquisition system.

This study successfully captured the 478 keV boron signal in solution, powder, and granular forms. Additionally, a detailed evaluation of the main background sources when using the CdTe‐DSD was conducted, and methods to improve image contrast were used. The irradiation times in these experiments ranged from 2 to 20 h, whereas a typical clinical BNCT treatment lasts only about 0.5 to 1 h. Because the neutron flux at the PGA facility is approximately one to two orders of magnitude lower than that of AB‐BNCT, this extended irradiation was necessary to ensure sufficient statistical precision under low‐flux conditions. However, in AB‐BNCT environments with higher neutron energies, we need to improve the detector efficiency due to the lower 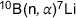 reaction rate. Based on the experimental results, we identified several necessary improvements for clinical AB‐BNCT applications. We plan to implement the following three upgrades to the detection system.

First, the 2‐mm‐thick CdTe crystal used in this experiment exhibited low photoelectric absorption efficiency (less than 5%) for 478 keV γ‐rays.^39^, ^40^ Because the detection sensitivity for 478 keV γ‐rays strongly depends on the CdTe thickness, we plan to develop a thicker CdTe‐DSD with a thickness of 5–10 mm to improve detection efficiency. We will also investigate a stacked configuration (e.g., two 5‐mm‐thick detectors) to achieve higher efficiency,^41^ while considering potential effects on background and charge spreading.

Second, because the CdTe‐DSD was originally developed for space observation, the data acquisition (DAQ) system is not optimized for high‐intensity quantum beam environments. In this experiment, the CdTe‐DSD had a dead time exceeding 50%. Such high dead time limits the operation of the CdTe‐DSD under AB‐BNCT conditions with higher beam intensities. Therefore, we have designed a new DAQ system with 10 times higher speed for quantum beam experiments and plan to validate its performance in future tests.

Finally, a 2‐mm‐diameter pinhole collimator was placed 158.5 mm from the sample and 48 mm from the detector in this experiment. The pinhole collimator maintained high spatial resolution and reduced geometric blurring even when placed at a distance from the sample. This spatial localization capability was confirmed by a sample‐shift test using the point‐like 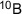 samples under neutron irradiation, as shown in Figure S4. However, the long distance between the sample and the collimator significantly reduced the number of γ‐rays entering the pinhole. As a result, the detection efficiency was lowered. To address this, we plan to adopt a parallel‐hole collimator with 1‐mm‐diameter holes to enhance sensitivity without compromising spatial resolution. Since the spatial resolution of parallel‐hole collimators is affected by the distance between the collimator and the sample, we will position the collimator as close as possible to the sample to ensure spatial resolution and sufficient sensitivity in future experiments. This configuration is expected to reduce acquisition time, making the system more suitable for real‐time imaging of larger target areas. With these three improvements, we expect a significant enhancement in the detection sensitivity for the 478 keV signal.

The background contribution is expected to be higher in AB‐BNCT environments due to the higher neutron flux. Although the background contributions under AB‐BNCT conditions cannot be estimated from the experimental results at PGA, our results have demonstrated that improving neutron shielding can effectively enhance CNR performance. Therefore, a new neutron shielding system using 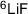 tiles will be implemented to suppress neutron‐induced interference around the detector system.

To further assess the clinical applicability of the CdTe‐DSD, we plan to conduct an experiment that replicates AB‐BNCT treatment conditions. A head phantom containing water and 50–100 ppm of 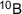 will be prepared to simulate human tissue. Experiments will be conducted using a higher‐energy neutron source in the few keV range to evaluate both the impact of hydrogen‐induced prompt γ‐ray background at 2223 keV and the CdTe‐DSD's capability to image low‐concentration boron distributions under these conditions. These tests will more accurately assess the performance of the optimized CdTe‐DSD in realistic AB‐BNCT environments.

## 5 CONCLUSIONS

A high‐resolution imaging system using a CdTe‐DSD was successfully demonstrated for real‐time 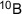 measurements in BNCT. The CdTe‐DSD is capable of effectively detecting the distribution of 478 keV prompt γ‐rays emitted from the 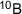 during neutron irradiation at the PGA. The results demonstrate the feasibility of detecting 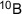 distribution with a spatial resolution of 4 mm in various sample forms, including solutions, powders, and granular boron. This study also investigated the effect of neutron shielding using LiF tiles, demonstrating that the use of LiF tiles considerably reduces the background from 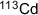 prompt γ‐rays. By comparing sample No. 7 in Experiment 3 with sample No. 2 in Experiment 1, both having an activity level of approximately 1 MBq, we observed a 14‐fold enhancement in the CNR. These findings suggest that under conditions of sufficiently effective neutron shielding, the CdTe‐DSD‐based SPECT system offers a promising tool for real‐time dosimetry in BNCT, enabling more accurate treatment planning and patient dose monitoring. Further improvements, including a new neutron shielding setup and a parallel‐hole collimator, will be pursued to evaluate the system's performance in clinical settings and its ability to visualize boron distribution in human tissues with low 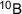 concentrations.

## CONFLICT OF INTEREST STATEMENT

The authors declare no conflicts of interest.

## Data Availability

The data supporting this study are available upon request from the corresponding author.
